# The lifetime prevalence of hospitalised head injury in Scottish prisons: A population study

**DOI:** 10.1371/journal.pone.0210427

**Published:** 2019-01-17

**Authors:** T. M. McMillan, L. Graham, J. P. Pell, A. McConnachie, D. F. Mackay

**Affiliations:** 1 Institute of Health and Wellbeing, University of Glasgow, Glasgow, United Kingdom; 2 Information Services Division, NHS National Services Scotland, Edinburgh, United Kingdom; Texas Technical University Health Sciences Center, UNITED STATES

## Abstract

**Background:**

There is mounting evidence that associates brain injury and offending behaviour, and there is a need to understand the epidemiology of head injury in prisoners in order to plan interventions to reduce associated disability and risk of reoffending. This is the first study to determine the lifetime prevalence of hospitalised head injury (HHI) in a national population of current prison inmates. In addition characteristics of prisoners with HHI and were compared to prisoners without HHI to discover whether those with HI differed demographically.

**Methods:**

Whole life hospital records of everyone aged 35 years or younger and resident in a prison in Scotland on a census date in 2015 were electronically linked via their unique NHS identifier and checked for ICD-9 and 10 codes for head injury. Using a case-control design, these data were compared with a sample from the general population matched 3:1 for age, gender and area-based social deprivation. Comparison of demographic variables was made between prisoners with and without HHI.

**Results:**

HHI was found in 24.7% (1,080/4,374) of prisoners and was significantly more prevalent than found in the matched general population sample (18.2%; 2394/13122; OR 2.10; 95%CI 1.87, 2.16). The prevalence of HHI in prisoners and controls was similar with the exception of a higher risk of HHI in prisoners in lower deprivation quintiles. Having three or more HHI was more common in prisoners (OR 3.04; 95%CI 2.33, 3.97) as were HHI with ICD codes for intracranial injuries (OR 1.81; 95% CI 1.54, 2.11), suggesting that more severe HHI is more prevalent in prisoners than the general population. The distributions within demographic variables and the characteristics of HHI admissions in prisoners with and without a history of HHI were similar.

**Conclusion:**

Prisoners in Scotland aged 35 years or younger have a higher lifetime prevalence of HHI than the general population and are more likely to have had repeated HI or intracranial injuries. Further work is required to elucidate the correspondence between self-report of HI and hospitalised records and to ascertain persisting effects of HI in prisoners and the need for services to reduce associated disability and risk of reoffending.

## Introduction

Meta-analyses estimate the lifetime prevalence of head injury (HI) in prisoners to be 50–60% [1,2]. This high lifetime prevalence could imply that HI in prisoners is a major issue in terms of health service need. The relationships between severe HI, cognitive impairment and personality change are known to be associated with neurobehavioural effects, including impulsiveness, impatience, intolerance, impaired insight, lack of concern for others, impaired concentration and memory, poor planning and problem solving, aggression and social disinhibition [3,4]. There is mounting evidence to suggest that these effects of HI lead to risk taking and breaking social rules that can lead to involvement with the criminal justice system, challenging behaviour in prison and recidivism [5–7].

In the general population more than 90% of HI are mild, and long term disability is not expected [8]. If this pattern is replicated in prison populations, then the estimates of a high lifetime prevalence of HI in prisoners could lead to a considerable over-statement of health service need. A recent systematic review assessed the risk of methodological bias in prevalence studies on HI in prisoners as being high overall, and highlighted several weaknesses, including that all previous studies are based on self-report of HI, there is little appropriate comparison with non-prisoners, none provide national coverage and in fact, most rely on samples that are not, or are not shown to be, representative of the prison population [9]. The social and economic costs of imprisonment are high, and it has been argued that brain injury in prisoners is likely to make a significant contribution to these costs [10], and that there is a clear need to establish a service pathway for those with persisting effects of HI, and in order to do this effectively, further epidemiological work is needed [11].

The present study is unique in that it is the first to describe the lifetime prevalence of hospitalised head injury (HHI) in a national (Scotland) population of current prison inmates and compares this to matched controls from the general population in Scotland. The prison population with HHI is further defined by comparison with the general population with HHI and the prison population without HHI. The study links ICD codes with the population of prisoners in Scotland on a census date. ICD codes fall under a heading of injury or injury to the head and do not themselves define traumatic brain injury. Although for some sub-headings it is clear that a traumatic brain injury has occurred (eg intracranial injury) for others it is not (eg ‘concussion’ or ‘unspecified injury to the head’). The term “head injury” does not make the assumption of brain injury and is used here to be mindful that not all of those reporting an injury to their head may have sustained a brain injury.

## Methods

This study employed a case-control design to compare the population with HHI in prison with a comparison group with HHI from the general population in Scotland who were matched for age, gender and deprivation. Within-group analysis compared those with and without HHI in the prison population. Prisoners were on remand or convicted of a crime and were incarcerated in a prison; those in custody in a police station were excluded.

An arbitrary census date (7th August 2015) was chosen for all inmates in Scottish prisons. All prisoners received into Scottish prisons have a National Health Service (NHS) health assessment, and all with a residence in Scotland have a unique NHS identifier, the Community Health Index (CHI), [12]. The CHI for each prisoner was extracted from the prison based health records and then electronically linked to Scottish Morbidity Records-01 (SMR-01) which are generated for all admissions to acute (non-obstetric, non-psychiatric) hospitals in Scotland. SMR-01 records the dates of admission and discharge and codes diagnoses using the International Classification of Diseases (ICD-9 from 1980 to 1996 and ICD-10 thereafter). Although there seems to be some consistency across studies in their use of ICD-9 codes for traumatic brain injury, there is not consistency in use of ICD-10 codes [13]. In the present study, codes that are likely to signify extracranial injury or facial fracture were excluded. The codes used to identify HI for prisoners and controls were as follows: ICD-9 800, 801; 803, 804 and 850–854; ICD-10 SO2.0, S02.1, S02.3, S02.7-S02.9, S06.0-S06.90 and S09.90. Codes designated by ICD as intracranial injury (ICD-9 851–854; ICD-10 S06.1-S06.9) with the exception of concussion, were used as indicators of ‘more severe’ HI, with concussion excluded because this can often typify a mild injury. Diagnosis of concussion often relies on subjective (self-report) symptom complaint by the patient and does not have a basis in brain scanning unlike the other ICD codes for intracranial injury [14].

As SMR-01 dates from 1980, inclusion in the study was restricted to prisoners aged 35 years or younger at the census date to ensure whole life coverage of all prisoners in the study. This age group is of particular interest given the long established heightened risk of offending and reoffending in younger adults [15,16].

In this way, information was extracted from SMR-01 on occurrence of HHI, duration of admission, age at HHI and time since each HHI was obtained in addition to age at census, gender and postcode. Postcode of residence was used to derive area social deprivation using the Scottish Index of Multiple Deprivation [17], (SIMD 2012). The latter was categorised into general population quintiles.

The comparison group from the Scottish general population (excluding current prisoners), was generated using a unique NHS identifier (Community Health Index), matched for age (date of birth), gender and SIMD quintile with three controls for each prisoner and then linked to SMR-01 from which data on HHI was extracted. We provide demographic data on the prisoner population and the Scottish general population for comparison here and potentially in future studies on other national populations.

Record linkage was undertaken by the NHS Information Services Division and the data analysed in the NHS National Safe Haven for Scotland by an investigator. The National Safe Haven allows data from electronic records to be used to support research when it is not practicable to obtain individual patient consent, while protecting patient identity and privacy.

Associations are reported as odds ratios (OR) and 95% confidence intervals (95% CI). To assess whether HHI is associated with being a prisoner, we treated being a prisoner as the outcome (case) variable, and used conditional logistic regression to account for the matched nature of the data, with HHI as the only covariate. This model was then extended to test for interactions between HHI and each of the three matching variables. Conditional logistic regression was also used to assess whether the risk of becoming a prisoner was associated with the age at HI, number of HI admissions, or length of admissions for HI (with no HHI as the reference group). We included these three variables because of interest in this field about whether earlier age at injury is a predictor of offending and because a greater number HI or longer admissions are likely to be associated with more severe HI [18,19]. For analyses that looked at comparisons within the prisoner population, we used ordinary logistic regression to estimate the OR and 95% CI for associations between HHI and age, sex, and SIMD quintile.

## Approvals

Permission was obtained from the Caldicott Guardian Committee of the Scottish Government whose remit includes regulation of the use and transfer of patient-identifiable information between NHS organisations and non-NHS bodies (Ref: 2015–18) and from the NHS Scotland Privacy Advisory Committee (Ref 71/14). Consent was not obtained from individuals and was not required as all information provided to the study was anonymised.

## Results

On the census date there were 8,010 prisoners in prisons in Scotland and data linkage between CHI and SMR-01 was achieved in 7,687 (96.0%). The remainder could not be linked because the postcode held by the prison was the prison itself (eg if homeless) or they did not reside in Scotland prior to imprisonment. Of those linked, 4,374 (56.9%) were aged 35 years or younger and comprise the prisoner population in the study. Data were missing for the first 4.75 months of the lives of 47 prisoners aged 35; this had a negligible effect on the overall prevalence of HHI found in prisoners (under-estimate by approximately 0.006%; see S1 Text). A matched comparison group of 13,122 was identified from the Scottish general population (Table 1). Demographic information on the prison population for all ages is given in S1 Table.

**Table 1:** Demographic characteristics of prisoners, matched comparison group and entire Scottish general population aged 16–35 years.

		Prisoners N (%)	Matched general population Comparison Group N (%)	Entire Scottish general population N (%)
**Gender**	Male	4,126 (94.3)	12,378 (94.3)	689,952 (49.6)
	Female	248 (5.7)	744 (5.7)	700,837 (50.4)
**Age category**	16–25	1,537 (35.1)	4,611 (35.1)	687,673 (49.4)
	26–35	2,837 (64.9)	8,511 (64.9)	703,116 (50.6)
**Deprivation**^1^	1 (high)	2,405 (55.0)	7,215 (55.0)	295,060 (21.2)
	2	1,004 (23.0)	3,012 (23.0)	286,774 (20.6)
	3	555 (12.7)	1,665 (12.7)	283,845 (20.4)
	4	290 (6.6)	870 (6.6)	259,638 (19.1)
	5 (low)	120 (2.7)	360 (2.7)	265,472 (19.1)
**Total**		4,374	13,122	1,390,787

^1^Scottish Index of Multiple Deprivation 2012 [17]

### Prisoners and the general Scottish population

The majority of the prison study population were male, in the 26–35 year age band and from the most deprived areas in Scotland. In comparison to the entire Scottish general population in the same age range, prisoners were more likely to be male, aged 26–35 years and to come from the most deprived quintile.

### Hospitalised head injury in prisoners and in the matched general population comparison group

The overall lifetime prevalence of HHI in the prisoner population aged 35 years or younger was 24.7% (n = 1080/4374) and this was significantly higher than the 18.2% (n = 2394/13122) found in the matched general population comparison group. HHI was more prevalent in prisoners than in the comparison group, in both male and female prisoners, in the 16–25 and in the 26–35 age ranges and in all deprivation quintiles (Table 2). The distribution of HHI in males and females or by age band was similar to that in the comparison group, whereas HHI tended to be more common in SIMD quintiles 2–5 in prisoners.

**Table 2:** Demographic features of prisoners and the matched general population group (total). Comparisons are between those with hospitalised head injury (HHI) in each group (Odds Ratio, 95% Confidence Interval and p for interaction).

	Prisoners		Non Prisoners		Univariate Odds Ratio 95% CI	P for interaction
	Total	N (% HHI)	Total	N (% HHI)		
All	4,374	1,080(24.7)	13,122	2,394(18.2)	2.10 (1.87, 2.16)	
Male	4,126 (94.3)	1,034 (95.7)	12,378(94.3)	2,316 (96.7)	2.07 (1.84, 2.33)	
Female	248 (5.7)	46 (4.3)	744 (5.7)	78 (3.3)	3.07 (1.77, 5.35)	0.170
Age 16–25	1,537 (35.1)	304 (28.1)	4,611 (35.1)	678 (28.4)	1.98 (1.60, 2.44)	
Age 26–35	2,837 (64.9)	776 (71.9)	8,511 (64.9)	1,716 (71.7)	2.16 (1.88, 2.48)	0.498
SIMD1 (high)	2,405 (55.1)	602 (55.7)	7,215 (55.0)	1,617 (67.5)	1.34 (1.15, 1.56)	
SIMD2	1,004 (22.7)	252 (23.3)	3,012 (23.0)	456 (19.0)	3.28 (2.56, 4.21)	
SIMD3	555 (12.7)	124 (11.5)	1,665 (12.7)	201 (8.4)	3.66 (2.59,5.18)	
SIMD4	290 (6.8)	71 (6.6)	870 (6.6)	87 (3.6)	5.73 (3.52, 9.33)	
SIMD5 (low)	120 (2.8)	31 (2.9)	360 (2.7)	33 (1.4)	6.46 (3.08, 13.59)	<0.001

OR from conditional logistic regression; SIMD, Scottish Index of Multiple Deprivation

The first admission with HHI was more common in the prisoner group than in the comparison group for age bands above 5 years, one or more admissions and by duration of admission (Table 3).

**Table 3:** Characteristics of hospital admissions for head injury in prisoners and the general population comparison group.

	Prisoners with HHI N (%)	Non Prisoners with HHI N (%)	Univariate Odds Ratio 95%CI
**Age at First Admission (years)**			
No HI	3,294 (75.3)	10,728 (81.8)	1.0
<1	48 (1.1)	141 (1.1)	1.28 (0.87, 2.06)
1–5	227 (5.2)	723 (5.5)	1.02 (0.28, 1.27)
6–10	202 (4.6)	504 (3.8)	1.83 (1.45, 2.31)
11–15	175 (4.0)	396 (3.0)	1.68 (1.33, 2.14)
16–20	216 (4.9)	339 (2.6)	3.81 (3.09, 4.70)
21–25	143 (3.3)	213 (1.6)	3.89 (3.02, 5.02)
26–35	69 (1.6)	78 (0.6)	6.58 (2.26, 9.66)
**Number of Admissions**			
No HI	3,294 (75.3)	10,728 (81.8)	1.0
1	673 (15.4)	1,641 (12.5)	1.75 (1.52, 2.01)
2	231 (5.3)	456 (3.5)	2.61 (2.06, 3.31)
3 or more	176 (4.0)	297 (2.3)	3.45 (2.62, 4.45)
**Length of Stay**			
No admission	3,294 (75.3)	10,728 (81.8)	1.0
1 to 2 days	979 (22.4)	2,202 (16.8)	2.05 (1.81, 2.31)
3 days or more	101 (2.3)	192 (1.5)	2.8 (1.96, 3.99)

OR from conditional logistic regression

The lifetime prevalence of ‘more severe’ HI as designated by ICD codes for intracranial injures excluding concussion, was higher in prisoners (8.6%; 378/4374)) than in the comparison group (7.0%; 914/13122; OR 1.51; 95% CI 1.28, 1.79). If taking individuals with three or more hospitalisations for HI not coded as ‘more severe’ as having potentially sustained cumulative effects that may increase the risk of persisting impairment [20,21], ‘more severe’ HI in these terms was also more common in prisoners (4.0%; 175/4374) than in the general population sample (2.1%; 280/13122; OR 3.04; 95% CI 2.33, 3.97). Finally, if considering those who either had intracranial injuries or three or more HHI as being at risk for persisting impairment, this risk was higher in prisoners (10.9%; 476/4374) than in the comparison group (8.1% 1,066/13122; OR 1.81; 95% CI 1.54, 2.11).

### Prisoners with and without hospitalised head injury

Within the prisoner population, those with HHI were more often male and aged 26–35 and did not differ by deprivation quintile (Table 4).

**Table 4:** Demographic features of prisoners with and without hospitalised head injury (HHI).

	Prisoners with HHI N (%)	Prisoners without HHI N (%)	Univariate Odds Ratio 95% CI
Male	1,034 (25.1)	3,092 (74.9)	1.51 (1.09, 2.10)
Female	46 (18.5)	202 (81.5)	1.0
Age 16–25	304 (19.8)	1,233 (80.2)	1.0
Age 26–35	776 (27.4)	2,061 (72.6)	1.53 (1.31, 1.77)
SIMD1 (high)	602 (25.0)	1,803 (75.0)	1.0
SIMD2	252 (25.1)	752 (74.9)	1.00 (0.85, 1.19)
SIMD3	124 (22.3)	431 (77.7)	0.86 (0.69, 1.07)
SIMD4	71 (24.5)	219 (75.5)	0.97 (0.73, 1.29)
SIMD5 (low)	31 (25.8)	89 (74.2)	1.04 (0.69, 1.59)

OR from ordinary logistic regression; SIMD Scottish Index of Multiple Deprivation

## Discussion

Studies in the general population in Sweden, Finland, Australia, New Zealand and Canada suggest that imprisonment or criminal convictions are more prevalent in the general population in people with a history of HI than in people without [22–26]. Where siblings with and without HI are compared, the effect persists suggesting that there could be a link that is substantially independent of genetics or environment [22]. This is the first study to provide lifetime data on a current prison population in an entire country and uses routine administrative data to provide objective data on HI with matching to a general population comparison group.

There are two main findings, and each points to a need for further research. The first, is that the overall lifetime prevalence of HHI was high in prisoners aged 35 or younger, being found in almost a quarter, with the risk of a prisoner having had a HHI being higher than in a matched sample from the general population. This lifetime prevalence is lower than the 50–60% estimated in meta-analyses of studies on adult prisoners using self-report [1,2]. These meta-analyses comprise studies that are entirely based on self-report where lifetime prevalence ranges from 10 to 100% in adults. The meta-analyses do not use quality ratings and the individual studies are subject to methodological bias in relation to lifetime prevalence estimates [9]. The accuracy of self-report or of records of hospitalisation as a true estimate of lifetime prevalence remains an issue [27]. Although records indicating hospitalisation are likely to be accurate, little work has been published on relationships between self-report of HI and corroboration in hospital records. In a cross-sectional study, Schofield et al [28] reported that of 112 prisoners who self-reported a history of HI, there was evidence for hospital attendance in 70%. The discrepancy may in part be explained by offenders not always attending hospital after a HI; for example if acute effects of HI are mistaken for effects of intoxication with drugs or alcohol, or if the HI was sustained during criminal activity. Alternatively prisoners may not remember their history of HI accurately or may not have attended hospital if the HI was perceived to be minor. Further work is needed to elucidate this. It seems though, that studies on prisoners using self-report, estimate a higher prevalence of HI than is indicated by hospital records.

A further issue in relation to the lower lifetime prevalence found in the present study than from the estimates in the meta-analyses is age. The present study was restricted to lifetime data for prisoners aged 35 or younger and it might be that prevalence of HHI is much higher in older prisoners. Few studies report lifetime prevalence stratified by age, although one study suggests that this is not the case. Colantonio et al [29], observed that 58% of prisoners aged under 35 years self-reported a history of HI, compared to 41% aged over 34 years. Overall, this does point to a need for further work to elucidate whether there are ages where risk of HI is particularly high in prisoners as this may inform programmes designed to educate prisoners about the risks, causes and consequences of HI. Prisoners in the lowest deprivation quintiles had a higher risk of HHI than the general population. The reason for this is not clear, but might reflect either a greater likelihood of prisoners who are less deprived attending hospital after a HI than prisoners who are more deprived, or that less deprived prisoners are exposed to greater risk than their counterparts in the general population.

The second main finding is that ‘more severe’ HI seems more common in prisoners than in the matched comparison group. It is difficult to classify severity of HI with certainty using ICD codes, and the assumption here is that intracranial injury is likely to represent moderate-severe HI and that repeat HI (three or more not coded as intracranial injury) is likely to have a cumulative effect. If using the occurrence of either of these as a proxy measure of moderate-severe HHI, 10.9% of prisoners aged 35 years or younger were hospitalised with a ‘more severe’ HI. The importance of this finding is in relation to whether there is likely to be persisting effects of HI and the implications for service delivery [9]. If prisoners had largely suffered a single mild HI, where good recovery without disability is generally expected [8] then arguably there may be little need to consider routine screening for HI in prisons or the provision of interventions, but with more severe or repeat HI comes risk of persisting disability. Findings here suggest that there may be a need to screen for a history of HI in prisoners in order that they might be triaged to appropriate services which might range from group based education to intensive neurorehabilitation [11]. These interventions might facilitate a change in behaviour and reduce the risk of future HI and potentially of reoffending in addition to reducing disability associated with previous HI. As ICD codes are not ideal for classification of severity of HI, these findings need to be corroborated in studies using established assessments of HI severity and outcome in order to estimate the lifetime prevalence of disability resulting from HI and hence service need in prisoners [7,11]. Duration of length of hospital stay might be considered to be a further indicator of severity of injury, with admission for more than 48 hours more likely to be severe, as found in 2.3% of admissions in prisoners with HHI in the present study.

Demographically prisoners with and without HHI were similar, both having a preponderance of men and coming from more deprived backgrounds, although the proportion of men was exaggerated further in the HHI group. This is generally consistent with demographic studies on HI in the general population [30,31]. Both men and women prisoners were at greater risk of HHI than the general population. Women comprise a small proportion of the prison population (5.7% in the present study) and perhaps for this reason have tended to receive less attention than men in studies on HI in prisoners. One study noted a higher occurrence of a history of physical and sexual abuse in women than in men in prison with self-report of HI [29]. However, the extent to which HI occurred in the context of psychological or physical abuse or the extent to which HI needs to be accounted for in management or interventions for mental health problems associated with such abuse is not known and further work is needed.

## Strengths and limitations

This is the only published study investigating HI in an entire national prison population. A high linkage between the prison population and health records was achieved. The study also benefits from comparison with a matched sample from the general population. A limitation is the absence of hospital health records prior to 1980, although the lifetime prevalence of HHI in prisoners aged 35 and under is provided. Furthermore, younger prisoners are of particular interest, given their relatively high risk of reoffending [16]. The study is also limited by consideration only of hospitalised HI and not also self-report. Finally, it is possible that some in the general population comparison group could have been ex-prisoners and we could not ascertain this.

## Conclusions

Prisoners in Scotland aged 35 or younger had a higher lifetime prevalence of HHI than a matched sample from the general population. They were more likely to have repeated HI and intracranial injuries more serious than concussion. Further work is required to elucidate the relationship between self-report of HI and hospital records and to ascertain persisting effects and the need for education aimed towards reducing the risk for repeat HI and for services to reduce associated disability and risk of reoffending.

## Supporting information

S1 Text Missing data. (DOCX)

S1 Table Demographic information for prisoners, and the entire Scottish population for ages 16–79. (DOCX)
