# Supplementary material for: The lifetime prevalence of hospitalised head injury in Scottish prisons: A population study
Source: PLoS One. 2019 Jan 17;14(1):e0210427. doi: 10.1371/journal.pone.0210427 (PMC6336306; doi:10.1371/journal.pone.0210427)
Supplement: S1 Text — (DOCX) [file pone.0210427.s001.docx]

**Supplementary File: S1 Text**

Missing Data: For the 98 prisoners who were born in 1979, SMR-01 records were not available for the first 4.75 months of their life; of these 41 were later admitted with a HHI and hence, already contribute to the overall HHI prevalence figure of 24.69%. If some of the remaining 57 ***only ever*** had one HHI in these missing 4.75 months and had a risk of doing so at the same rate as in the remaining population of prisoners in the first year of their lives (see table 3) there would be up to one prisoner with undetected HHI.

That is, 1.1% of prisoners had their first HI in their first year of life (see table 3). Hence, with data missing for 57 prisoners at risk of HI for 4.75 months of time, that equates to approximately 22.5 years at risk. There would be an expected 0.25 people with an unobserved HHI event during that period. That would add an expected 0.0057% to the prevalence of HHI in the prisoners. The missing data has a negligible effect and would not alter the rounded figure of 24.7% for the prevalence of HHI in prisoners given in the paper.
