# Supplementary material for: The lifetime prevalence of hospitalised head injury in Scottish prisons: A population study
Source: PLoS One. 2019 Jan 17;14(1):e0210427. doi: 10.1371/journal.pone.0210427 (PMC6336306; doi:10.1371/journal.pone.0210427)
Supplement: S1 Table — (DOCX) [file pone.0210427.s002.docx]

**Supplementary File: S1 Table**

**S1 Table: Demographic information for prisoners, and the entire Scottish population for ages 16-79**

|  |  | **Prisoners**  **N (%)** | **Entire Scottish Population aged 16-79**  **N (%)** |
| --- | --- | --- | --- |
| **Gender^1^** | Male | 7,260 (94.4) | 2,050,430 (48.7) |
|  | Female | 427 (5.6) | 2,160,160 (51.3) |
| **Age band^1^** | 16-25 | 1,537 (20.0) | 689,210 (16.4) |
|  | 26-35 | 2,837 (36.9) | 705,953 (16.8) |
|  | 36-45 | 1,864 (24.3) | 677,380 (16.1) |
|  | 46-55 | 999 (13.0) | 800,885 (19.0) |
|  | >=56 | 450 (5.9) | 1,337,162 (31.8) |
| **Social Deprivation^2^** | 1 (high) | 4,106 (53.4) | 788,364 (18.8) |
|  | 2 | 1,790 (23.3) | 814,337 (19.4) |
|  | 3 | 921 (12.0) | 859,033 (20.5) |
|  | 4 | 545 (7.1) | 877,900 (21.0) |
|  | 5 (low) | 250 (3.3) | 84,9854 (20.3) |
|  | Not known | 75 (1.0) | -- |

^1^Statistics for mid-2015, Scottish Government; ^2^SIMD 2012 quintiles
